# Biogeography and assembly processes of abundant and rare soil microbial taxa in the southern part of the Qilian Mountain National Park, China

**DOI:** 10.1002/ece3.11001

**Published:** 2024-02-13

**Authors:** Zhilin Feng, Na Li, Yanfang Deng, Yao Yu, Qingbo Gao, Jiuli Wang, Shi‐long Chen, Rui Xing

**Affiliations:** ^1^ Northwest Institute of Plateau Biology Chinese Academy of Sciences Xining Qinghai China; ^2^ College of Life Science University of Chinese Academy of Sciences Beijing China; ^3^ Service Center of Qilian Mountain National Park in Qinghai Province Xining Qinghai China; ^4^ Qinghai Provincial Key Laboratory of Crop Molecular Breeding Xining Qinghai China; ^5^ Qinghai Nationalities University Xining Qinghai China

**Keywords:** deterministic processes, Qilian Mountain National Park, Qinghai‐Tibet Plateau, rare bacterial taxa, rare fungal taxa, stochastic process

## Abstract

Soil microorganisms play vital roles in regulating multiple ecosystem functions. Recent studies have revealed that the rare microbial taxa (with extremely low relative abundances, which are still largely ignored) are also crucial in maintaining the health and biodiversity of the soil and may respond differently to environmental pressure. However, little is known about the soil community structures of abundant and rare taxa and their assembly processes in different soil layers on the Qinghai‐Tibet Plateau (QTP). The present study investigated the community structure and assembly processes of soil abundant and rare microbial taxa on the northeastern edge of the QTP. Soil microbial abundance was defined by abundant taxa, whereas rare taxa contributed to soil microbial diversity. The results of null model show that the stochastic process ruled the assembly processes of all sub‐communities. Dispersal limitation contributed more to the assembly of abundant microbial taxa in the different soil layers. In contrast, drift played a more critical role in the assembly processes of the rare microbial taxa. In addition, in contrast to previous studies, the abundant taxa played more important roles in co‐occurrence networks, most likely because of the heterogeneity of the soil, the sparsity of amplicon sequencing, the sampling strategy, and the limited samples in the present study. The results of this study improve our understanding of soil microbiome assemblies on the QTP and highlight the role of abundant taxa in sustaining the stability of microbial co‐occurrence networks in different soil layers.

## Graphical abstract

The present study investigated community structure and assembly processes in the southern part of Qilian Mountain National Park.

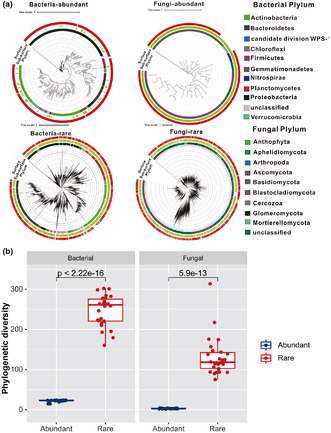

## 1 INTRODUCTION

The Qinghai‐Tibet Plateau (QTP) is the highest plateau (average elevation above 4000 m) in the world and is the largest in China. The Qilian Mountain National Park (Qinghai) is located on the northeastern edge of the QTP, covering an area of 50,200 km^2^; the main land cover types are desert grassland and alpine meadow, and forests (*Picea crassifolia* forest and Qilian cypress forest) are mainly located along the eastern edge. The average elevation is above 3000 m, making the park a crucial ecological barrier for deserts from the northern and eastern sides. In addition, unlike the platform of the QTP, the area is located in the convergence zone of the monsoon and westerlies and is thus more sensitive to the monsoon climate (Yan & Ding, 2020).

Soil microorganisms constitute an essential part of the biodiversity in the soil and play vital roles in regulating multiple ecosystem functions in terrestrial ecosystems (Luo et al., 2020). Therefore, studies on the diversity and composition of soil microbial communities and their biogeographic patterns are necessary to reveal the details of such ecological processes (Charlop‐Powers et al., 2015; Liu et al., 2019). The large‐scale distribution patterns of soil microbes are currently well documented because of the development of high‐throughput sequencing technology. Relevant studies indicate that soil microbial communities are dominated by a few microbial phyla. Regarding the soil bacterial community at a global level, the phyla Actinobacteria, Proteobacteria, and Acidobacteria are dominant, and nearly 2% of the bacterial phylotypes account for almost half of the abundance. Previous studies indicated that soil pH plays an important role in shaping the soil microbial community (Delgado‐Baquerizo et al., 2018; Zeng et al., 2019). Egidi et al. (2019) revealed that regarding the global distribution pattern of soil fungi, Ascomycota and Basidiomycota are the most abundant fungal phyla, and soil pH and mean annual precipitation are the main factors driving the fungal community (Egidi et al., 2019). Later research confirmed that the global soil fungal community is dominated by Ascomycota (Aslani et al., 2022). In our previous studies, we investigated the microbial communities in the Qaidam Basin, which is adjacent to the Qilian Mountain National Park (Qinghai), and found that a few phyla dominated both soil bacterial and fungal communities. Local environmental factors and spatial distance shaped the bacterial community, and the influence of spatial distance on the fungal community was weak (Xing et al., 2019, 2021).

Most previous studies on the distribution patterns of soil microbial communities focused on the abundant members (with higher relative abundances), mainly because it is assumed that they play important roles in various ecological processes (Bahram et al., 2015; Griffiths et al., 2016). However, with continuous research and the development of various techniques, recent studies have revealed that the rare microbial taxa (with extremely low relative abundances, e.g., <0.01%) are also important in maintaining the health and biodiversity of the soil and may respond differently to environmental pressure (Saleem et al., 2016). For instance, genome sequencing of the rare, uncultured bacterial phylum TM7 revealed its essential roles in ecology and evolution (Albertsen et al., 2013). A study on the crop microbiomes showed that Sordariomycetes and Dothideomycetes dominated the fungal community, but the diversity was mainly ensured by rare taxa, which play a more important role in ecosystem functioning and key biochemical soil processes (Xiong et al., 2021).

In peatland, *Desulfosporosinus* (with a relative abundance of 0.006%) plays an important role as a sulfate reducer (Pester et al., 2010). It is generally accepted that rare taxa respond differently to environmental changes than abundant taxa. For example, during the chemical stabilization of cadmium (Cd)‐contaminated soil, a narrower niche breadth to soil pH and Cd was observed for rare bacterial taxa compared to abundant ones, indicating that physical and chemical properties are more important in shaping the communities of rare bacterial taxa (Xu et al., 2021). In paddy soils, environmental factors (such as pH, elevation, and geographic distance) differently affect the abundant and rare sub‐communities in China (Hou et al., 2020), and similar results have been reported for other soil ecosystems (Chen, Xing, et al., 2019; Fierer & Jackson, 2006; Lauber et al., 2009). However, our knowledge about the mechanisms that sustain the biodiversity of rare taxa and their important ecological functions in different ecosystems is still limited.

In recent years, research about microbial assembly processes (species into communities) has become an important topic in microbial ecology studies. Those processes are comprised of deterministic and stochastic processes (Stegen et al., 2013), of which the first are controlled by biotic and abiotic factors, which include environment filtering (such as soil pH and nutrient level) and biological interactions (Fargione et al., 2003). Stochastic processes include random changes such as birth, death, dispersal limitation, and ecological drift (Chave, 2004). The null model analysis based on phylogenetic and taxonomic β‐diversity metrics has been used to evaluate the balance between those two processes (Stegen et al., 2013), and the assembly mechanisms of soil abundant and rare taxa have been widely documented. However, the effects of deterministic and stochastic processes on soil abundant and rare taxa vary with different habitats. For example, for agricultural soil, the abundant taxa were driven by stochastic processes, whereas the rare ones were driven by deterministic processes (Jiao & Lu, 2020). Similarly, in coastal wetlands, rare taxa are more significantly governed by deterministic processes, whereas abundant ones are more influenced by stochastic processes (Gao, Peng, et al., 2020). In contrast, in rice paddy soils across China, deterministic processes dominate the abundant bacterial taxa, whereas stochastic processes dominate the rare ones (Hou et al., 2020). In addition, the assembly processes of soil bacterial and fungal (abundant and rare taxa) communities show various responses to environmental changes because of the differences in dispersal and nutrient use (Zheng et al., 2021). For example, in a study on orchard soil, the abundant and rare fungal taxa were largely influenced by stochastic and deterministic processes, respectively, whereas the abundant and rare bacterial taxa were most impacted by deterministic processes (Zhao et al., 2022). Thus, to understand the effects of deterministic and stochastic processes on microbial community assembly processes, both bacterial and fungal soil communities need to be investigated.

More and more studies compare the community structures of surface and subsurface soil microbes, mainly because soil microbes of the deeper soil layers also play an important role in various environments (Fierer et al., 2003; Hartmann et al., 2009). So far, most studies in this field have revealed differences in the microbial communities for surface and subsurface soil layers due to the different soil physical and chemical properties. Generally, bacterial and fungal diversity decreases with increasing soil depth (Chen, Saleem, et al., 2019), although the opposite pattern has also been observed. For example, Mueller et al. (2015) found that soil fungal communities were significantly different among different soil layers, and operational taxonomic unit (OTU) richness was higher in the subsurface than in the surface soil layer (Mueller et al., 2015).

The soil microbial communities of different layers in the QTP are well studied. Research in the permafrost regions on the QTP has shown that Acidobacteria, Proteobacteria, and Bacteroidetes dominate the bacterial communities, and distinct bacterial communities were found among the different layers (0–10, 10–20, and 20–30 cm) (Wu, Xu, et al., 2017). In another study, the soil microbial communities in the high‐elevation desert on the QTP differed between the surface (0–15 cm) and subsurface (15–30 cm) soil layers. Soil physical and chemical properties, climate factors, and geographic distance are the most suitable predictors for both bacterial and fungal communities (Chu et al., 2016). Studies on the soil microbial community assembly processes in different soil depths on the QTP are scarce. However, because of the different characteristics of assembly processes of abundant and rare taxa, such studies are crucial (Peng et al., 2021).

Here, we investigated the community structure and assembly processes of abundant and rare taxa from different soil layers (0–15 and 15–30 cm) of 13 sites in the Qilian Mountain National Park (Qinghai). The average elevation was above 3000 m, and the largest distance between sites was 491 km. We hypothesized that the microbial communities differ in their structure between the different soil layers and for abundant and rare taxa. We also assumed that the assembly processes differ between abundant and rare taxa and between the different soil layers and that there are differences between bacteria and fungi, caused by differences in resource use and adaptation to environmental changes.

## 2 METHODS

### 2.1 Soil sampling and analysis of soil physical and chemical properties

**FIGURE 1:**
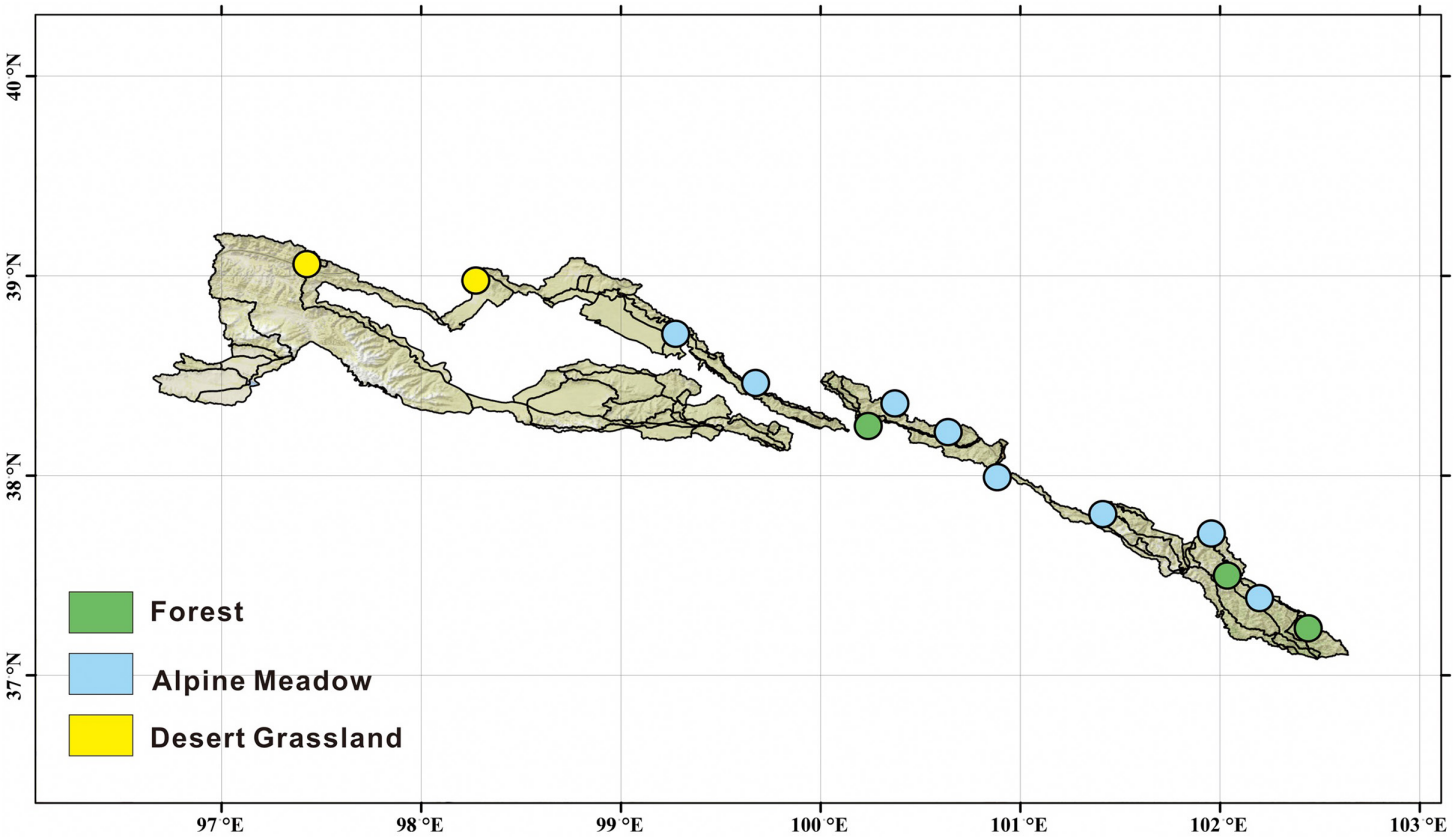
The Qilian Mountain National Park (Qinghai) and the distribution of sampling sites.

The 13 sampling sites were located on the northeastern edge of the QTP, in the Qilian Mountain National Park (Qinghai), Qinghai Province, China (Figure 1 and Table S1). The main vegetation types are alpine meadow (eight sites), desert grassland (two sites), and forest (three sites). The average distance between sites was 13–491 km, and the average elevation was 3320 m (2480–4097 m). In 2020, at each site, mixed soil samples from five 100‐m^2^ plots were taken from two soil layers (surface: 0–15 cm, subsurface: 15–30 cm) and passed through a 2‐mm screen for subsequent sequencing and chemical analysis. The methods of determining soil pH, total soil phosphorus (TP), total carbon (TC), total potassium (TK), total nitrogen (TN), nitrate nitrogen (${\mathrm{NO}}_{3}^{-}$), ammonium nitrogen (${\mathrm{NH}}_{4}^{+}$), carbon:nitrogen ratio (C:N), and the soil moisture content (MC) are described in our previous study (Xing et al., 2018). Climate data (annual precipitation: AP, annual mean temperature: AMT) were obtained from the WorldClim database (www.worldclim.org).

### 2.2 DNA extraction and HiSeq sequencing

Total soil DNA was extracted from 0.5 g of each soil sample using the FastDNA SPIN Kit for Soil (MP Biomedicals, California), following the manufacturer's recommendations. The samples were then analyzed in Sangon Biotech Co., Ltd., Shanghai, China, using the primers 515F (GTG CCA GCM GCC GCG GTAA) and 909R (CCC CGY CAA TTC MTT TRA GT) (Liu et al., 2007) for bacteria and ITS1F (CTT GGT CAT TTA GAG GAA GTA A) and ITS2 (GCT GCG TTC TTC ATC GAT GC) (Gardes & Bruns, 1993) for fungi at the Illumina HiSeq2500 platform. The PCR products were purified using EasyPure Quick Gel Extraction Kits (TransGen Biotech, Beijing, China), and libraries (barcoded) were constructed with the TruSeq™ DNA Sample Prep Kit (Illumina, San Diego, CA). The sequence data were submitted to GenBank under the accession numbers SAMN27408966–SAMN27409017.

### 2.3 Bioinformatics

The tools USEARCH v. 10 (Edgar, 2010) and Qiime2 (Bolyen et al., 2019) were employed to analyze the amplicon data as follows: (1) primers and low‐quality sequences (shorter than 200 bp or with a quality score (*Q*) < 30) were removed and forward and reverse reads were merged into a single sequence by using USEARCH; (2) sequences were clustered into operational taxonomic units (OTUs) based on a 97% similarity cutoff; (3) the taxonomy of OTUs was analyzed by the RDP Classifier against the SIlVA (release 138; for bacteria) (Bolyen et al., 2019) and UNITE (v. 8.0; for fungi) databases, and the OTUs that did not belong to bacteria and fungi were removed before prior to analysis. The definition of abundant (with relative abundance >0.1%) and rare (with relative abundance <0.01%) microbial taxa groups followed the conception of former studies (Jiao & Lu, 2020; Zheng et al., 2021). We set the sampling depth to 10,000 for bacteria and to 5000 for fungi to remove samples with low sequence reads for these analyses.

### 2.4 Statistical analysis

The α‐diversity indices of microbial communities (abundant and rare communities) were calculated in R (http://www.r‐project.org) with the vegan package (Dixon, 2003), and one‐way analysis of variance (ANOVA) was used to test the significance of the differences between rare and abundant taxa, using the software package SPSS v. 26.0. The software STAMP (v. 2.1.3) was employed to evaluate the relative abundances of abundant and rare taxa for different groups, based on the Kruskal‐Wallis test (*p* < .01) (using the Benjamini‐Hochberg false discovery rate (FDR) correction) (Parks et al., 2014). The Bray–Curtis dissimilarity between different soil layers and between abundant and rare taxa of bacteria and fungi was calculated by using the verdict function in the “vegan” package. All sequences were aligned by the software MAFFT (v. 7.490) (Katoh & Standley, 2013), and the phylogenetic tree (maximum likelihood tree) was constructed by FastTree (Price et al., 2010) and visualized by iTOL (v. 5) (Letunic & Bork, 2021). Phylogenetic diversity was calculated based on the method described by Faith (1992), using the package picante in R (Faith, 1992). Levin's niche breadth index was used to evaluate the ecological adaptation of abundant and rare taxa, using the package spaa (Zhang & Ma, 2013) in R. The Mantel test was applied to evaluate the relationships among community dissimilarity (based on Bray‐Curtis community dissimilarity), geographic distance, soil physical and chemical properties, and climate factors in R. To measure the assembly processes of rare and abundant taxa in different soil layers, the Infer Community Assembly Mechanisms by Phylogenetic‐bin‐based null model (iCAMP) were used (the analysis was performed using the R software (4.2.1) “iCAMP” package). To quantify various ecological processes, the observed taxa are first divided into different groups (‘bins’) based on their phylogenetic relationships. Then, the process governing each bin is identified based on null model analysis of the phylogenetic diversity using beta Net Relatedness Index (βNRI), and taxonomic β‐diversities using modified Raup–Crick metric (RC). For each bin, the fraction of pairwise comparisons with βNRI < −1.96 is considered as the percentages of homogeneous selection, whereas those with βNRI > +1.96 as the percentages of heterogeneous selection. Next, the taxonomic diversity metric RC is used to partition the remaining pairwise comparisons with |βNRI| ≤ 1.96. The fraction of pairwise comparisons with RC < −0.95 is treated as the percentages of homogenizing dispersal, while those with RC > +0.95 as dispersal limitation. The remains with |βNRI| ≤ 1.96 and |RC| ≤ 0.95 represent the percentages of drift (Stegen et al., 2013, 2015). Based on the OTU level, the bacterial and fungal co‐occurrence networks in the different soil layers were analyzed using the online integrated Network Analysis Pipeline (iNAP, http://mem.rcees.ac.cn:8081/). Pair‐wise relationships among the OTUs were determined by SparCC analysis (Feng et al., 2022), at a SparCC correlation coefficient >.3 and a significance level of *p* > .05. The total number of edges (connections between nodes) and degrees of co‐occurrence (the number directly related to nodes) were used to deduce the network topology. The top 10 hubs with high degree and closeness centrality values were identified. Finally, the networks were visualized using Gephi (0.10.0) (Bastian et al., 2009).

## 3 RESULTS

**FIGURE 2:**
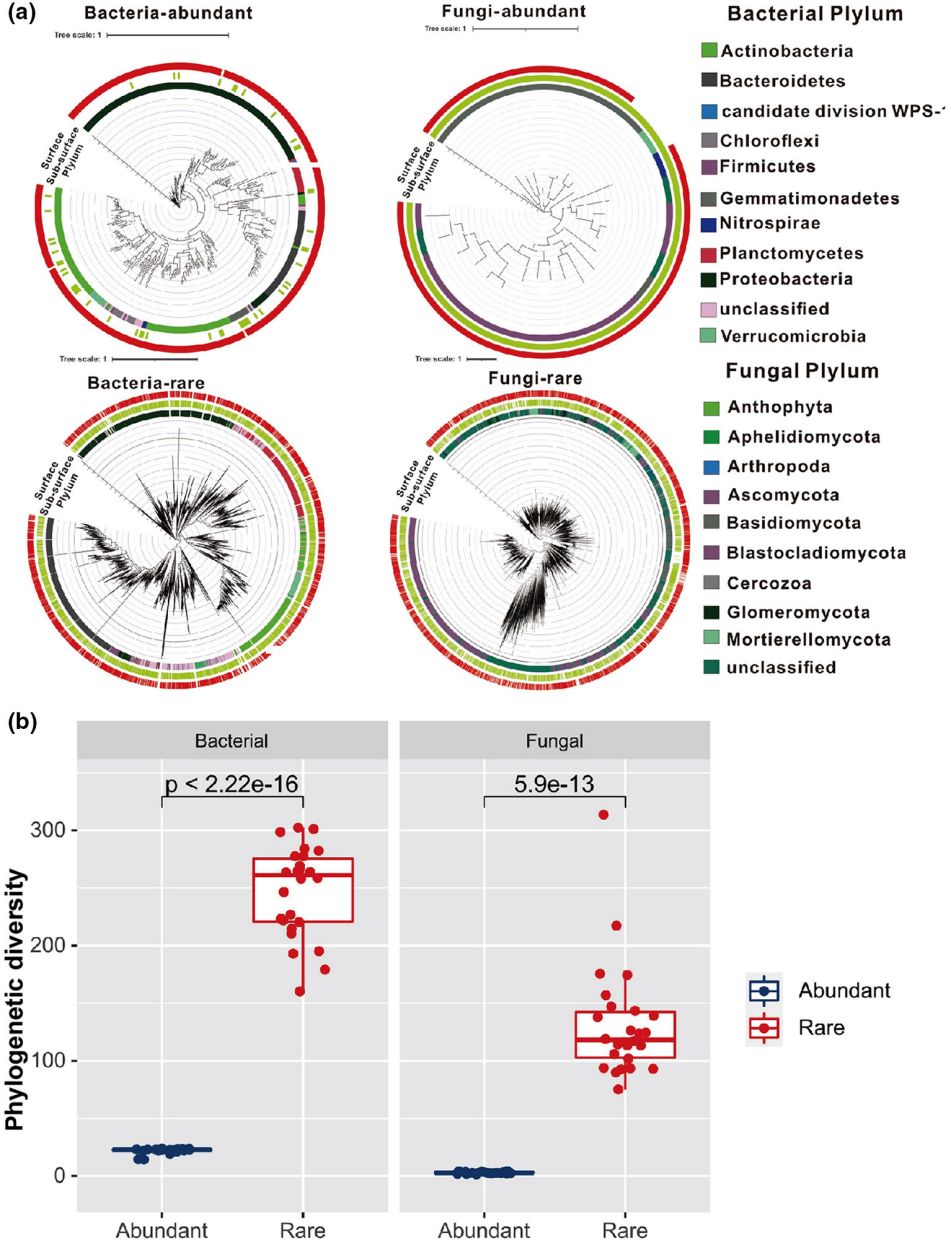
Phylogenetic tree and taxonomic composition of soil mycobiomes in the Qilian Mountain National Park (Qinghai). (a) Phylogenetic distribution of abundant taxa and rare taxa in different soil layers. (b) Difference of phylogenetic diversity between abundant and rare taxa.

A total of 2,098,946 and 2,439,413 bacterial and fungal reads were generated after removing the chimera and quality filtering, and 26,308 and 11,337 OTUs were obtained based on a 97% clustering threshold. For bacteria, only 164 abundant OTUs (0.62%) were generated, but they accounted for 50.6% of the relative abundance, and 24,230 rare bacterial OTUs (92.1%) accounted for 17.7% of the total relative abundance. Similarly, a higher proportion (57.2%) of rare fungal OTUs only accounted for 2.85% of the relative abundance, and a higher relative abundance of abundant fungal (27.9%) OTUs contributed only with 28 abundant fungal OTUs (0.25%) (Figure 2a and Figure S1).

**FIGURE 3:**
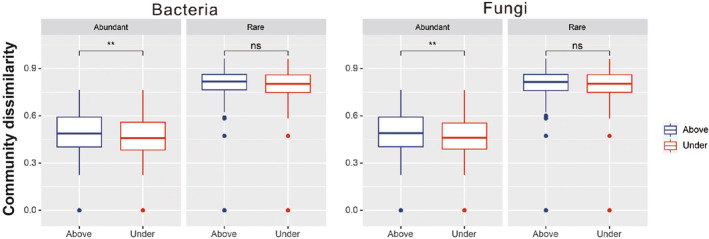
Community dissimilarity (based on Bray‐Curtis) of microbial ((a) Bacteria, (b) Fungi) abundant and rare taxa within different soil layers (0–15, 15–30 cm).

**FIGURE 4:**
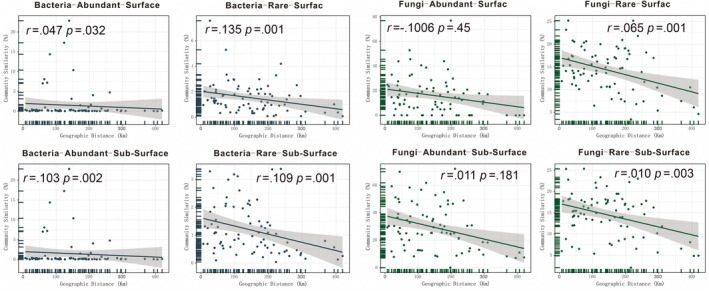
Relationship between community similarity and geographic distance (mantel test).

Proteobacteria, Acidobacteria, and Bacteroidetes were the most abundant bacterial phyla in all data sets (abundant and rare taxa) and both soil layers (Figure 2a and Figure S2). The phyla Cyanobacteria, Diapherotrites, Gemmatimonadetes, and Elusimicrobia were detected in the rare taxa but not in the abundant taxa (Figure 2a and Figure S2). In addition, in the rare taxa, the bacterial communities showed consistency between the different layers. For the abundant taxa, the relative abundances of Acidobacteria, Gemmatimonadetes, Bacteroidetes, Proteobacteria, and Latescibacteria significantly differed (*p* < .01) between the two soil layers (Figure S3). Regarding soil fungi, Ascomycota and Basidiomycota were the dominant phyla in all data sets. The rare taxa were similar in their community structure between the different soil layers, whereas the abundant taxa Ascomycota, Basidiomycota, Chytridiomycota, and Mortierellomycota significantly differed (*p* < .01) between the different soil layers (Figure S3). Closer phylogenetic relationships of abundant taxa both in bacteria and fungi were found (Figure 2a, Appendix S2), and the microbial phylogenetic diversity indices were also significantly lower than in the rare taxa, irrespective of the soil layer (Figure 2b, Appendix S2). For both bacteria and fungi, there were no significant differences in the ɑ‐diversity indices (Chao1, ACE, Observed, Shannon, and Simpson indices) of abundant and rare taxa between the different soil layers (Table S2). Soil depth had a stronger influence on the community dissimilarity (based on the Bray‐Curtis distance) of abundant than of rare taxa, and the community similarities of abundant bacterial and fungal taxa significantly differed between the two soil layers (Figure 3). The niche breadth index was significantly higher for abundant bacterial taxa than for rare taxa (*p* < .01); however, there were no significant differences between abundant and rare fungal taxa (Figure S4, Appendix S2). The Mantel test was used to evaluate the relationship between community dissimilarity and geographic distance. Rare bacterial and fungal taxa from both soil layers showed a significant (*p* < .01) distance decay. In contrast, only abundant bacterial taxa in the subsurface showed a significant (*p* < .01) distance decay (Figure 4).

**FIGURE 5:**
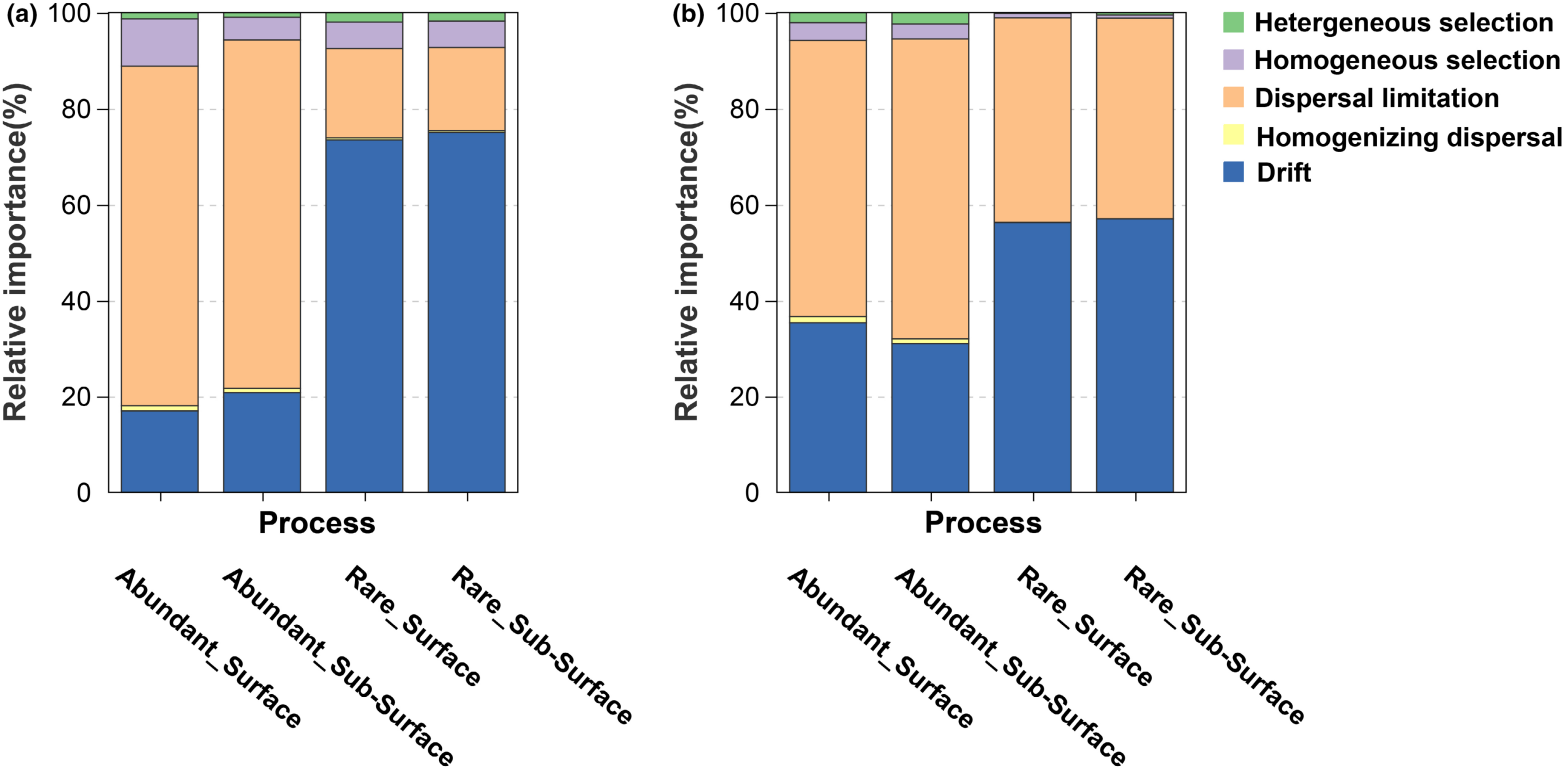
Assembly processes of rare and abundant microbial taxa.

**FIGURE 6:**
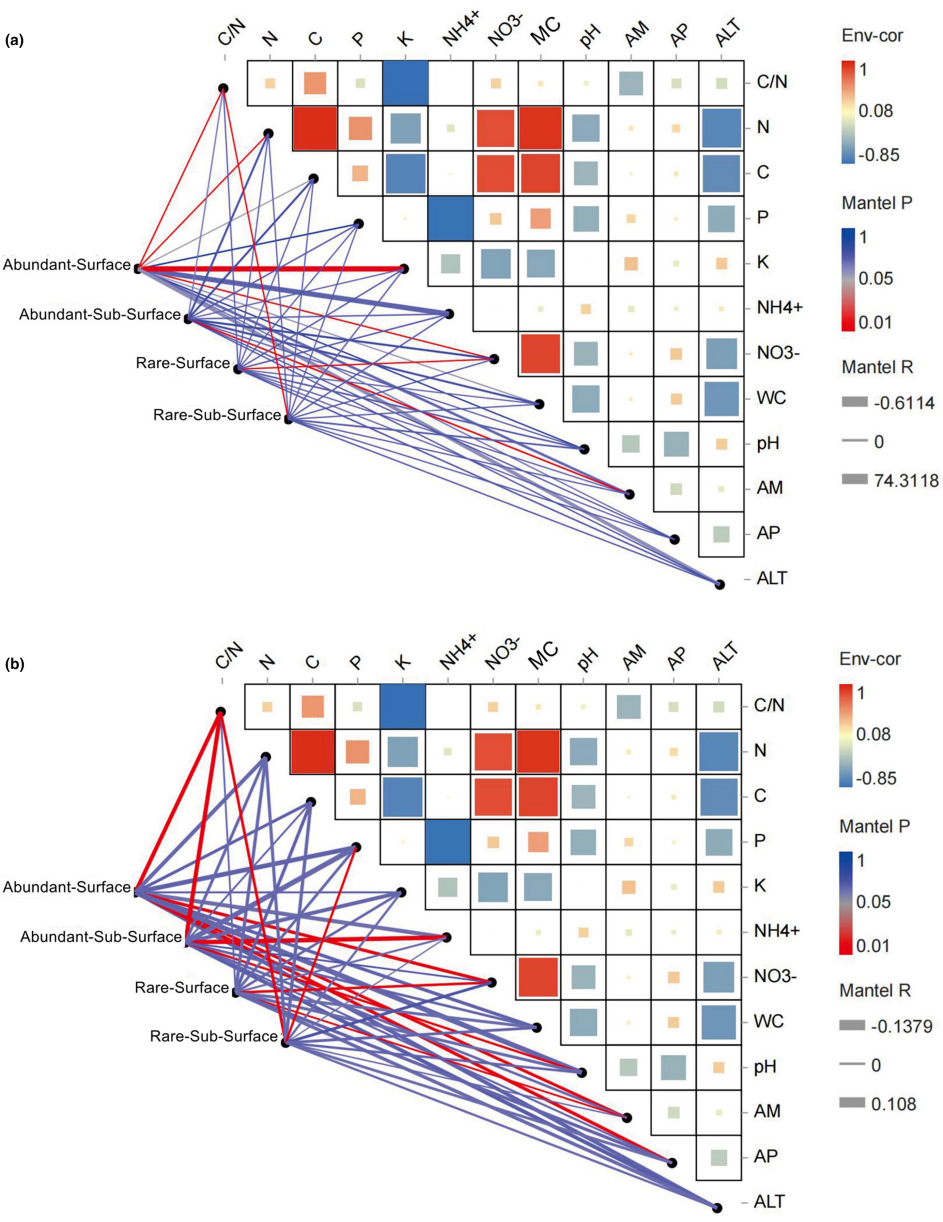
Correlations between microbial communities, soil physicochemical characteristics, and environmental factors. Different microbial communities (abundant and rare) from different soil layers (0–15, 15–30 cm) were related to each environmental factor by the Mantel test.

**FIGURE 7:**
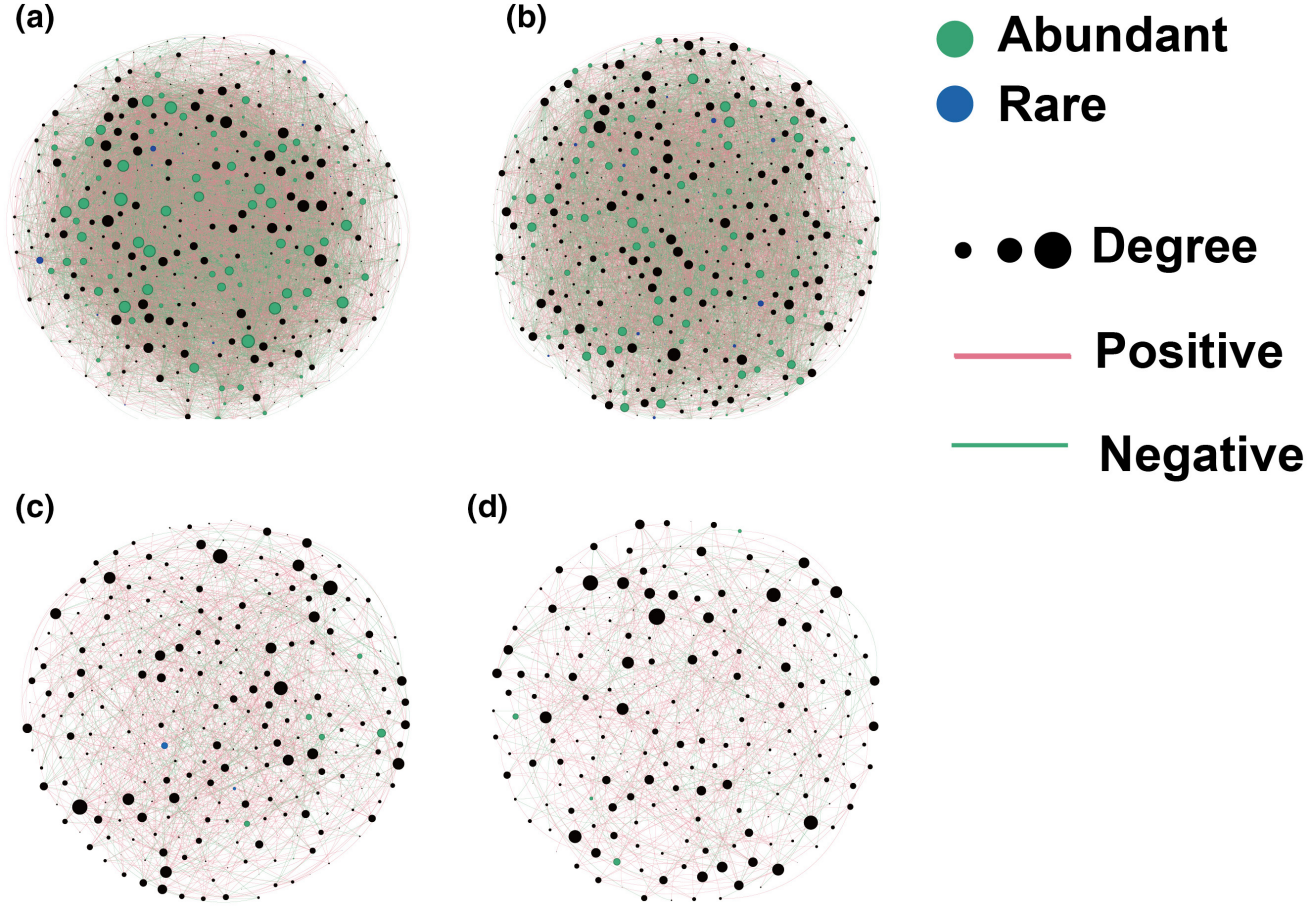
The microbial co‐occurrence networks in the different soil layers (a, bacterial surface network, b, bacterial sub‐surface network, c, fungal surface network, d, fungal sub‐surface network). Blue and green nodes represent rare and abundant OTUs. Node size is proportional to the number of connections (degree).

The results of null model show that the stochastic process ruled the assembly processes of all sub‐communities. Dispersal limitation contributed more to the assembly of abundant microbial taxa in the different soil layers. In contrast, drift played a more critical role in the assembly processes of the rare microbial taxa (Figure 5a,b). The results of the Mantel test suggest that the abundant bacterial taxa were more sensitive to environmental factors. For example, the rare bacterial taxa in the different soil layers were only regulated by one environmental factor (C/N and NO^3−^ for surface subsurface soil layers), but the surface bacterial abundant taxa were regulated by soil C/N, N, K, and ${\mathrm{NO}}_{3}^{-}$. In addition, environmental factors played a more important role in shaping the communities of abundant fungal taxa than those of rare taxa (Figure 6a,b and Table S3) in the two soil layers. In the microbial co‐occurrence networks analysis, all data sets were included (abundant, rare, and remaining taxa). For the bacterial networks, 967 and 1088 nodes were found in the surface and subsurface samples. Among them, in the surface soil samples, 128 abundant nodes (13.2%) and 137 rare nodes (14.2%) were identified (Figure 7a and Table S4), whereas in the subsurface soil samples, 137 rare (12.6%) and 156 abundant (14.3%) nodes were found (Figure 7b and Table S4). We identified 9736 and 6826 edges from the surface and subsurface soil networks, and the numbers of positive and negative edges among abundant nodes were consistent (for example, the numbers of positive edges both in the surface and subsurface soil networks were 718 (Figure 7a and Table S5) and 809 (Figure 7b and Table S5), respectively). The number of positive and negative edges between abundant and rare nodes was lower in the surface soil network (e.g., 70 positives were found in the surface soil network and 366 positive edges in the subsurface network) (Table S5). In addition, no edges between rare nodes were found in the surface soil bacterial networks, but we identified 723 and 625 negative edges from the subsurface soil bacterial networks (Table S5). The top 10 nodes with high degree and closeness centrality values were identified. In the surface soil network, 7 nodes were abundant nodes, observed for the phyla Bacteroidetes (3), Proteobacteria (2), Acidobacteria (1), and Chloroflexi (1), whereas only 1 surface soil rare node was found. Similarly, in the subsurface soil network, 6 abundant nodes (from the phyla Bacteroidetes (2), Acidobacteria (2), Chloroflexi (1), and Proteobacteria (1)) were found, but no subsurface soil rare node was detected (Table S6). For the fungal co‐occurrence networks, 588 and 454 nodes were generated from surface‐ and subsurface soil networks (Figure 7c,d). Among them, only a few nodes were identified as rare (six surface and four subsurface nodes) and abundant nodes (nine surface and six subsurface nodes) (Table S4). Consequently, the number of edges among abundant nodes was low (Table S5).

## 4 DISCUSSION

Understanding the species abundance distribution pattern in a community is important in ecological research. Generally, a few species make up an abundant species, along with many rare species (McGill et al., 2007). The present study shows the community structures and distribution patterns of abundant and rare soil microbial taxa from different soil layers in the northeastern part of the QTP. The soil microbial communities were dominated by a few OTUs (abundant taxa), and most OTUs (rare taxa) accounted for a small part of the total abundance. Although the abundance of rare taxa was extremely low, the phylogenetic diversity of rare taxa was significantly higher than that of abundant taxa. This finding is consistent with the results of our previous studies on the soil microbial communities from an adjacent area (the Qaidam Basin) (Xing et al., 2019, 2021) and agrees with the findings of other studies on agricultural soil (Hou et al., 2020; Zhao et al., 2022) as well as lakes and reservoirs. There are two potential explanations for the high relative abundances of a few microbial taxa. The first is the different effects of dispersal limitation on abundant and rare taxa. Rare taxa are more susceptible to dispersal limitation, thus showing a distance decay, whereas abundant taxa show a cosmopolitan distribution pattern over large geographic distances (Zhou & Ning, 2017). This is similar to the distribution pattens of large organisms (Grime, 1998), which may follow similar ecological rules. Abundant taxa (for example, the globally distributed soil fungal genera *Alternaria*, *Aureobasidium*, and *Cladosporium* and the cosmopolitan bacterial phyla Actinobacteria and Bacteroidetes) are usually the most abundant taxa in dust (Barberan et al., 2014), providing them with a higher mobility. Our results show that all rare microbial sub‐communities (surface and subsurface) showed significant (*p* < .01) distance decay, whereas only the abundant subsurface bacteria showed significant (*p* = .002) distance decay, which implies a stronger effect of dispersal limitation on rare microbes in the northeastern part of the QTP. Another possible reason is that, compared to abundant taxa, rare taxa have a weaker competition ability because of their lower resource use and weaker adaption to environmental changes, which restricts their distribution (Logares et al., 2015). In a previous study, abundant taxa showed broader response thresholds to environmental variables, and the niche breadths of microbes were influenced by the ranges of the environmental thresholds (Jiao & Lu, 2020).

Previous studies have found distinct microbial communities in different soil layers, which could be explained by differences in soil properties (Chu et al., 2016; Yang et al., 2017). More recently, other authors have shown that abundant microbes are more sensitive to soil depth (microbial alpha diversity and community composition of abundant taxa significantly differed among different soil layers) in the south of the Chinese Loess Plateau (Peng et al., 2021), which is similar to our finding that the microbial abundant sub‐community similarities (based on the Bray‐Curtis metric) were significantly different between surface and subsurface soil layers. In addition, the relative abundances of some abundant bacterial (Acidobacteria, Gemmatimonadetes, Bacteroidetes, Latescibacteria, and Proteobacteria) and fungal phyla (Ascomycota, Basidiomycota, Chytridiomycota, and Mortierellomycota) significantly differed (*p* < .01) between the two soil layers, most likely as a result of the different soil physical and chemical properties at each soil layer and the different effects of dispersal limitation on abundant and rare taxa in different layers.

No significant difference in alpha diversity was found between the different soil layers both for abundant and rare taxa. This could be explained by the extremely high diversity of rare taxa in all samples. A higher community diversity means that more individuals have various phenotypes and functions and that the community is more stable when facing environmental changes (Evans & Hofmann, 2012). Abundant communities were mainly composed of the same taxa (Proteobacteria, Acidobacteria, and Bacteroidetes for abundant bacterial taxa, Ascomycota and Basidiomycota for abundant fungal taxa), without significant differences in alpha diversity between the different soil layers.

There are two main theories regarding microbial community assembly processes. The niche theory considers that microbial communities are shaped by deterministic processes such as interspecies interactions and niche differentiation, whereas the neutral theory is based on the assumption that microbial communities are shaped by stochastic processes such as birth, death, and limited dispersal (Bahram et al., 2016). Both deterministic and stochastic processes can simultaneously influence microbial community assembly processes, and the balance between those two processes mainly depends on geographic scales and environmental gradients (Morrison‐Whittle & Goddard, 2015). Most studies in this field showed that the soil abundant taxa are mainly regulated by stochastic processes. Abundant taxa occupy a broader niche than rare taxa, have a higher capability of nutrient use and a higher growth rate and are thus more stable in a changing environment. By contrast, rare microbial taxa are mainly regulated by deterministic processes due to their narrow niche breadth, lower competitive ability, and lower growth rate (Gao, Peng, et al., 2020; Jiao & Lu, 2020; Xiong et al., 2021). Nevertheless, there are also inconsistent results. For example, studies in a rice field (Hou et al., 2020), in oil‐contaminated soil (Jiao et al., 2017), and in grassland soil on the QTP (Ji et al., 2020) showed that stochastic processes dominated the assembly of the rare taxa, whereas deterministic processes drove abundant taxa; the different results are mainly due to differences in habitat and geography (Shi et al., 2018). In the present study, the results of the null model analysis show that all the sub‐communities were dominated by stochastic processes (dispersal limitation plays a more important role in the abundant sub‐communities and drift plays a more important role in the rare sub‐communities). This result is consistent with the findings of previous studies in the northwestern Pacific Ocean (Wu, Logares, et al., 2017) and during successional reforestation (Peng et al., 2021). These results may be explained by the hypothesis proposed by Pandit et al. (2009) that taxa with a wide niche breadth may be primarily affected by dispersal limitation (Pandit et al., 2009). In addition, it has been shown that weak dispersal limitation allows drift to cause greater spatial turnover in community composition (Zhou & Ning, 2017).

The rare microbial communities in the different soil layers were mainly ruled by deterministic processes (drift). This is because rare taxa are more sensitive to environmental changes and therefore more likely to be stochastically pushed to local extinction (Sprockett et al., 2018). The different responses of rare bacterial and fungal taxa to soil depth were mainly caused by the different dispersal limitation effects on each sub‐community. Soil fungi and bacteria differ in cell size, which affects the dispersal ability and spatial aggregation of organisms and thus determines whether they are dominated by deterministic or stochastic processes (Jiao & Lu, 2020). In addition, several studies have shown that the effects of deterministic and stochastic processes are not immutable: for example, constantly changing soil organic matter levels can cause the transformation from deterministic to stochastic processes, and the influence of stochastic processes decreases during drought stress (Gao, Montoya, et al., 2020). This can partly explain the different responses of rare bacterial and fungal community assembly processes to different soil layers.

The microbial network was constructed based on microbial co‐occurrence microbial, each node in the network was a microbial OTU, and the edges between two nodes represent the significant association between these microbial OTUs. This approach not only facilitates the investigation of the potential interactions of microbial species but also could reveal potential ecological processes based on the high microbial diversity (Blanchet et al., 2020). Previous studies have investigated the microbial co‐occurrence network of abundant and rare taxa from different ecosystems. For example, Xiong et al. (2021) found that in the bacterial co‐occurrence network, the moderate and rare taxa play a more important role (with the highest numbers of nodes and edges) in maintaining the stability of the co‐occurrence network than abundant taxa (Xiong et al., 2021). Similarly, in orchard soil and in contaminated soil subjected to remediation (Xu et al., 2021), rare taxa have more nodes and a higher degree in the network (Zhao et al., 2022). However, in the present study, we found an opposite pattern. Although the moderate taxa dominated both bacterial and fungal co‐occurrence networks, in the microbial co‐occurrence networks, the numbers of abundant and rare nodes were similar. In addition, the average degree of abundant nodes was higher than that of rare nodes, irrespective of the soil layer. Most of the bacterial hub nodes were from abundant taxa, indicating that they play an important role in maintaining the stability of co‐occurrence networks. In addition, we found more positive and negative connections in the subsurface co‐occurrence networks between rare and abundant nodes and within rare nodes, indicating that rare taxa are more active in the subsurface soil. Regarding the fungal co‐occurrence networks, most nodes were from moderate taxa, suggesting that fungal abundant and rare taxa are not as important as previously assumed. This can be explained as follows: First, soil heterogeneity is a huge challenge in the construction of microbial co‐occurrence networks as it camouflages the differences in soil physicochemical properties among samples (Armitage & Jones, 2019), calling for more refined sampling methods (Raynaud & Nunan, 2014). Second, during data processing, rare taxa are filtered out due to their low prevalence (Cougoul et al., 2019). Third, amplicon sequencing is rarely performed because of the limited technical methods and the high soil microbial diversity (Kaul et al., 2017). In this context, greater sequencing depths and more samples are needed to obtain a comprehensive understanding of the soil microbial diversity.

This study investigated the community structure and assembly processes of abundant and rare microbial taxa in different soil layers of the northeastern edge of the QTP. Abundant taxa largely contributed to the soil microbial abundance, whereas rare taxa significantly contributed to diversity. Abundant and rare microbial taxa showed different responses to soil depth, but we found no significant difference in ɑ‐diversity between the different soil layers for abundant and rare microbial taxa. The abundant surface and subsurface microbial (bacteria and fungi) communities were dominated by deterministic processes, whereas the assembly processes of rare microbial taxa differed between different soil layers and between bacteria and fungi. They were largely caused by dispersal limitation and differences in the ability to use resources and adapt to environmental changes. Besides, in the co‐occurrence networks, abundant taxa may play a more important role as previously assumed. Our results reveal the fundamental soil microbial assembly processes in the southern part of the Qilian Mountain National Park and highlight the importance of abundant taxa in microbial co‐occurrence networks.

## AUTHOR CONTRIBUTIONS

**Zhilin Feng:** Data curation (equal); methodology (lead); resources (equal); software (equal). **Na Li:** Formal analysis (equal); software (equal). **Yanfang Deng:** Funding acquisition (equal); resources (equal). **Yao Yu:** Methodology (equal); visualization (equal). **Qingbo Gao:** Funding acquisition (equal); resources (equal). **Jiuli Wang:** Formal analysis (equal); validation (equal). **Shi‐long Chen:** Methodology (equal); project administration (equal); writing – review and editing (equal). **Rui Xing:** Project administration (lead); visualization (lead); writing – original draft (equal).

## CONFLICT OF INTEREST STATEMENT

None.

## Supporting information


Appendix S1.

Appendix S2.

## Data Availability

The sequence data were submitted to GenBank under the accession numbers SAMN27408966–SAMN27409017 (https://www.ncbi.nlm.nih.gov/bioproject/824613).
