# Supplementary material for: Biogeography and assembly processes of abundant and rare soil microbial taxa in the southern part of the Qilian Mountain National Park, China
Source: Ecol Evol. 2024 Feb 13;14(2):e11001. doi: 10.1002/ece3.11001 (PMC10862184; doi:10.1002/ece3.11001)
Supplement: Supplementary file 1 — Appendix S1. [file ECE3-14-e11001-s001.docx]

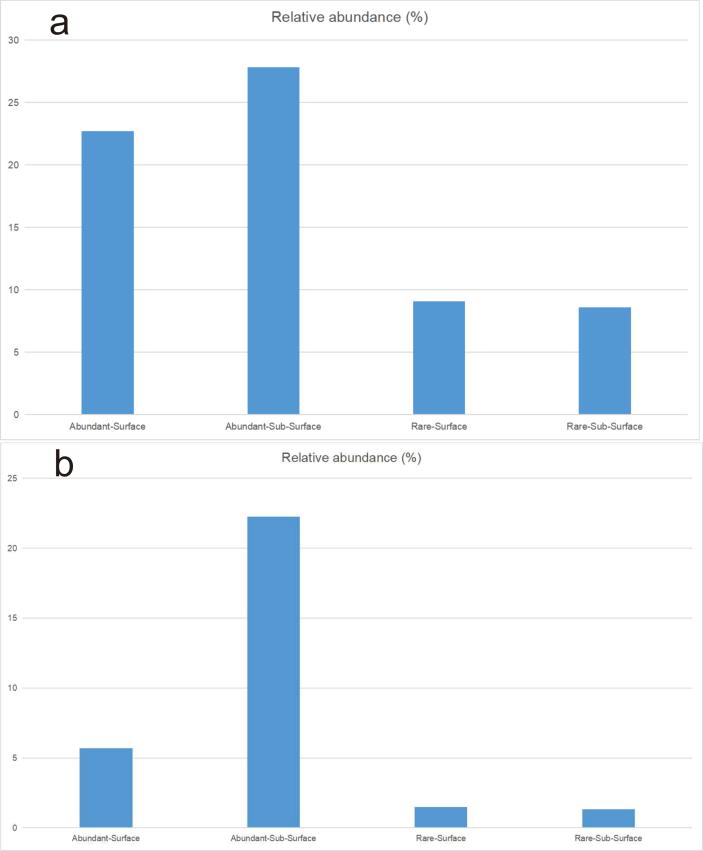


Fig.S1 The relative abundance of bacterial (a) and fungal (b) abundance and rare taxa in different soil layers (surface and sub-surface).


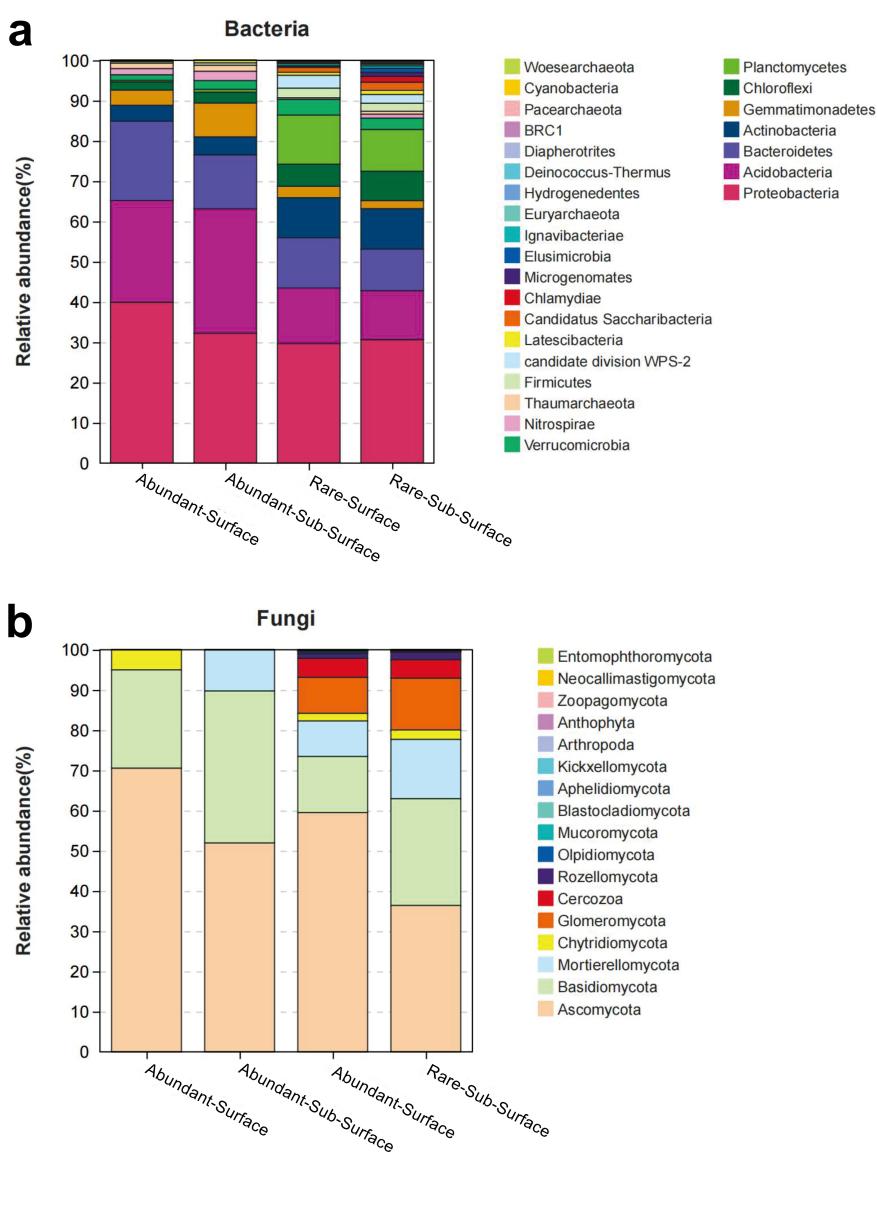


Fig. S2 Microbial community (a: bacteria, b: fungi) structure composition at the phylum level. Different microbial communities (abundant and rare) from different soil layers (0–15 cm15–30 cm) are presented.


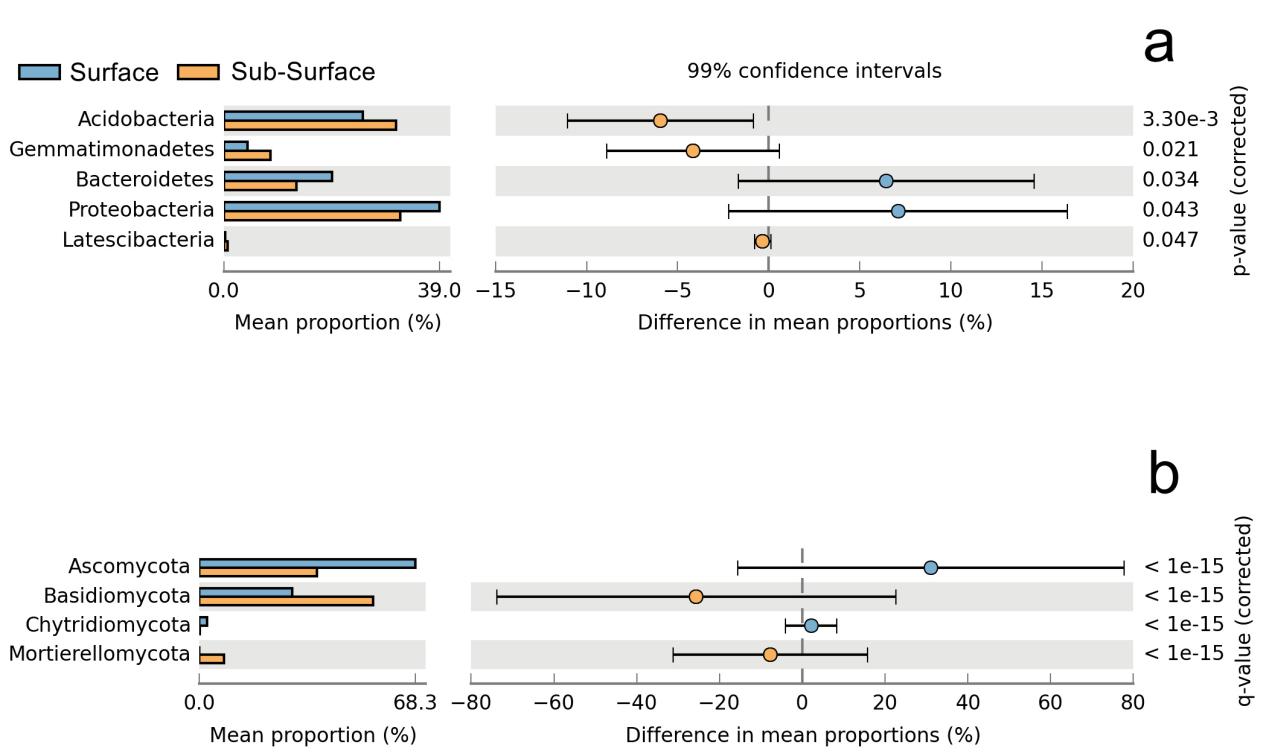


Fig. S3 Abundant bacterial (a) and fungal (b) phyla that were statistically significantly different (p < 0.01) between different soil layers (0–15 cm, 15–30 cm).


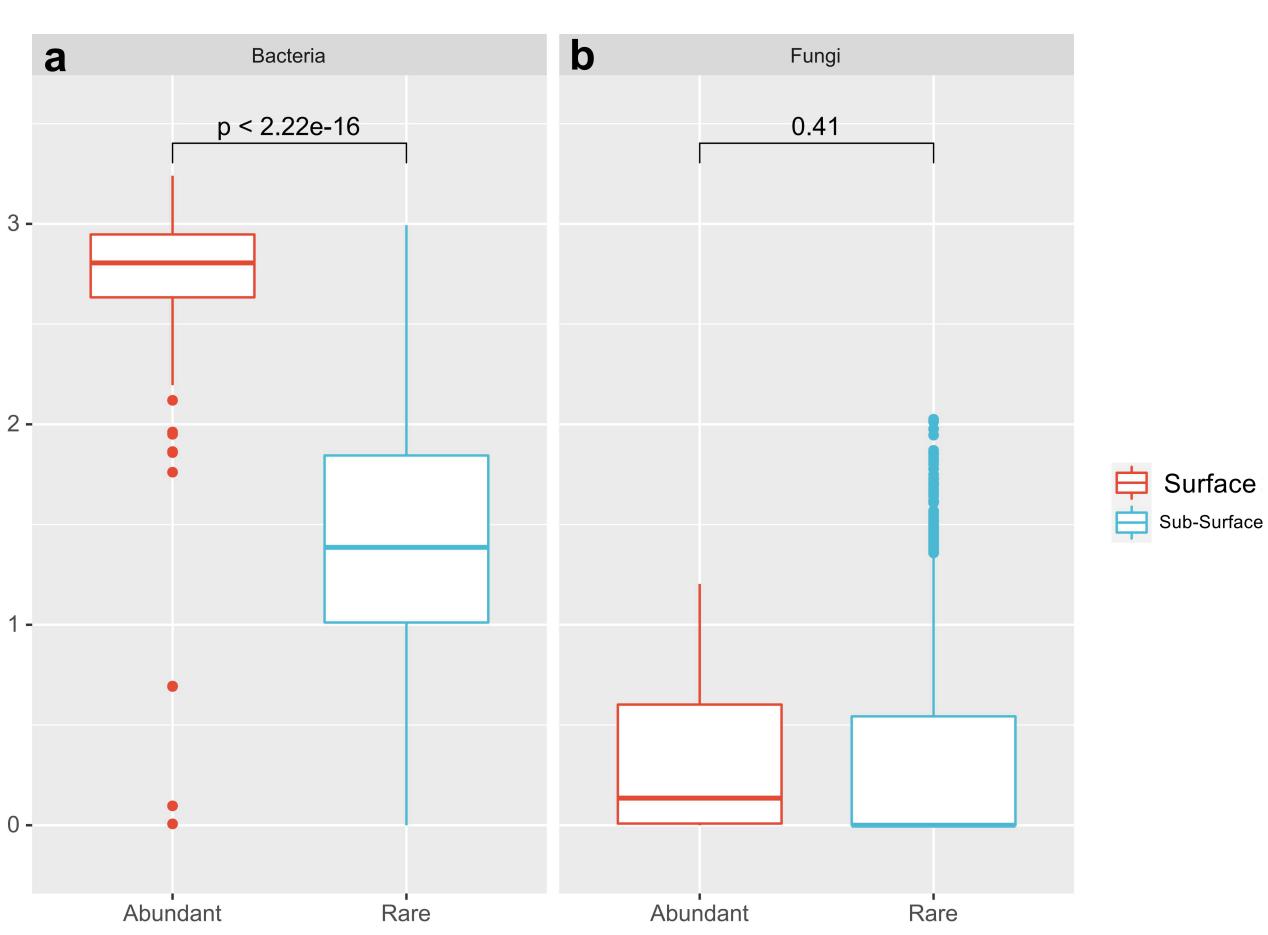


Fig. S4 Niche breadth index values for abundant and rare taxa of bacteria (a) and fungi (b).

Table s1 Sample information from 13 sites

| Sites | Sampling number | Lat. (°N) | Long. (°E) | ALT (m) | Ecological type |
| --- | --- | --- | --- | --- | --- |
| S1 | A1,U1 | 102°27′39.00″ | 37°07′58.57″ | 2480 | Forest |
| S2 | A2,U2 | 102°24′08.99″ | 37°18′29.22″ | 3711 | Alpine meadow |
| S3 | A3,U3 | 102°16′36.62″ | 37°19′56.64.22″ | 3245 | Forest |
| S4 | A4,U4 | 102°07′58.48″ | 37°38′48″ | 3120 | Alpine meadow |
| S5 | A5,U5 | 101°19′27.88″ | 37°47′11.26″ | 3640 | Alpine meadow |
| S6 | A6,U6 | 100°54'57.51″ | 37°59′56.10″ | 3664 | Alpine meadow |
| S7 | A7,U7 | 100°36'25.20" | 38°08'46.68" | 4005 | Alpine meadow |
| S8 | A8,U8 | 100°30'36.00" | 38°17'33.68" | 2799 | Forest |
| S9 | A9,U9 | 100°31'33.20″ | 38°09'00.32" | 3654 | Alpine meadow |
| S10 | A10,U10 | 38°16'49.05" | 99°51'59.84" | 3674 | Alpine meadow |
| S11 | A11,U11 | 100°28'55.92" | 37°47'47.40" | 3500 | Alpine meadow |
| S12 | A12,U12 | 98°26'84.92" | 38°94'95.00" | 3421 | Desert grassland |
| S13 | A13,U13 | 99°28'33.61" | 38°36'36.01" | 4097 | Desert grassland |

Lat.: latitude; Long.: Longitude; ALT: Altitude

Table S2 General description of the abundant and rare α-diversity index

| Category | Chao1 | ACE | Observed | Shannon | Simpson |
| --- | --- | --- | --- | --- | --- |
| Bacteria-Surface |  |  |  |  |  |
| Abundant taxa | 152.4 | 152.3 | 152 | 6.19 | 0.98 |
| Rare taxa | 6617.4 | 7089.1 | 3779 | 11.10 | 0.99 |
| Bacteria-Sub-Surface |  |  |  |  |  |
| Abundant taxa | 152.7 | 153.1 | 152 | 6.27 | 0.98 |
| Rare taxa | 6516.9 | 7018.1 | 3797 | 11.12 | 0.99 |
| Fungi-Surface |  |  |  |  |  |
| Abundant taxa | 7.76 | 9.25 | 7.31 | 1.25 | 0.46 |
| Rare taxa | 471.94 | 454.71 | 422.41 | 8.10 | 0.99 |
| Fungi-Sub-Surface |  |  |  |  |  |
| Abundant taxa | 9.02 | 11.89 | 7.31 | 0.97 | 0.39 |
| Rare taxa | 387.47 | 373.39 | 346.84 | 7.83 | 0.99 |

Table S3 Spearman’s correlations between microbial communities, soil physicochemical characteristics based on Mantel test

| Variables | | bacteria | | | | | | | | Fungal | | | | | | | | |
| --- | --- | --- | --- | --- | --- | --- | --- | --- | --- | --- | --- | --- | --- | --- | --- | --- | --- | --- |
|  |  | Surface | | | | Sub-Surface | | | | Surface | | | | Sub-Surface | | | | |
|  |  | Abundant | | Rare | | Abundant | | Rare | | Abundant | | Rare | | Abundant | | | Rare | |
|  |  | *r* | *P* | *r* | *P* | *r* | *P* | *r* | *P* | *r* | *P* | *r* | *P* | *r* | *P* | | *r* | *P* |
| Environmental  factors | ALT | -0.37 | 0.21 | 0.12 | 0.55 | -0.04 | 0.49 | 0.06 | 0.49 | 0.07 | 0.36 | -0.08 | 0.31 | 0.07 | | 0.39 | -0.04 | 0.32 |
|  | AM | -0.21 | 0.41 | -0.04 | <0.01 | 0.00 | 0.47 | -0.07 | 0.48 | -0.06 | <0.01 | 0.01 | <0.01 | -0.05 | | 0.33 | 0.00 | 0.35 |
|  | AP | 0.22 | 0.40 | 0.02 | 0.53 | 0.04 | 0.46 | 0.05 | 0.47 | -0.07 | <0.01 | 0.06 | 0.35 | -0.03 | | 0.35 | 0.04 | 0.30 |
|  | C | 0.54 | 0.12 | -0.11 | 0.59 | 0.04 | 0.49 | 0.06 | 0.53 | -0.06 | 0.37 | 0.05 | 0.36 | -0.02 | | 0.36 | 0.06 | 0.33 |
|  | C/N | 0.54 | <0.01 | -0.01 | 0.33 | 0.00 | 0.49 | -0.02 | 0.49 | 0.07 | <0.01 | 0.01 | 0.35 | 0.08 | | <0.01 | 0.04 | <0.01 |
|  | K | -0.61 | <0.01 | 0.04 | 0.46 | -0.02 | 0.48 | 0.06 | 0.48 | -0.02 | 0.38 | -0.05 | 0.34 | -0.09 | | 0.34 | -0.04 | 0.31 |
|  | N | 0.39 | <0.01 | -0.12 | 0.54 | 0.04 | 0.47 | -0.03 | 0.44 | -0.10 | 0.33 | 0.06 | 0.33 | -0.06 | | 0.34 | 0.05 | 0.32 |
|  | NH_4_^+^ | 74.31 | 0.43 | -0.03 | 0.47 | -0.03 | 0.50 | 0.03 | <0.01 | 0.08 | 0.29 | -0.03 | 0.32 | 0.08 | | <0.01 | 0.00 | 0.31 |
|  | NO_3_^-^ | 0.50 | 0.01 | -0.12 | 0.62 | 0.04 | <0.01 | 0.07 | 0.48 | -0.07 | <0.01 | 0.03 | <0.01 | 0.01 | | 0.44 | 0.04 | 0.45 |
|  | P | -0.02 | 0.89 | -0.04 | 0.46 | 0.04 | 0.48 | <0.01 | 0.50 | -0.12 | 0.36 | 0.04 | 0.33 | -0.14 | | 0.32 | 0.03 | <0.01 |
|  | pH | 0.06 | 0.78 | 0.06 | 0.49 | -0.06 | 0.47 | 0.02 | 0.50 | 0.11 | 0.34 | -0.06 | 0.31 | 0.03 | | <0.01 | -0.04 | 0.35 |
|  | MC | 0.37 | 0.21 | -0.12 | 0.56 | 0.05 | 0.45 | -0.02 | 0.49 | 0.07 | 0.36 | 0.05 | 0.31 | -0.05 | | 0.35 | 0.04 | 0.34 |

Table S4 The node number of the the networks

|  |  | Number of nodes | | Average degree | |
| --- | --- | --- | --- | --- | --- |
|  |  | Above | Under | Above | Under |
| Bacterial | All | 967 | 1088 | 41 | 26 |
|  | Abundant | 128 | 131 | 60 | 35 |
|  | Rare | 137 | 156 | 35 | 27 |
| Fungi | All | 588 | 454 | 7 | 5 |
|  | Abundant | 6 | 4 | 5 | 6 |
|  | Rare | 9 | 6 | 9 | 2 |

Table S5 Connection number of the networks.

|  |  | Total  connections | | Positive connections | | Negative connections | |
| --- | --- | --- | --- | --- | --- | --- | --- |
|  |  | Above | Under | Above | Under | Above | Under |
| Bacterial | Abundant-Abundant | 1530 | 1549 | 718 | 809 | 812 | 741 |
|  | Abundant-Rare | 184 | 795 | 70 | 366 | 114 | 429 |
|  | Rare-Rare | 0 | 1348 | 0 | 723 | 0 | 625 |
| Fungi | Abundant-Abundant | 1 | 0 | 1 | 0 | 0 | 0 |
|  | Abundant-Rare | 1 | 0 | 1 | 0 | 0 | 0 |
|  | Rare-Rare | 1 | 0 | 1 | 0 | 0 | 0 |

Table S6 Top 10 bacterial nodes with highest degree and closeness centrality values in the different soil layers

| Above |  | phylum | class | order | family | genus |
| --- | --- | --- | --- | --- | --- | --- |
| Otu6461 | abundant | Chloroflexi | unclassified | unclassified | unclassified | unclassified |
| Otu6462 | abundant | Proteobacteria | Gammaproteobacteria | Xanthomonadales | Xanthomonadaceae | Arenimonas |
| Otu6617 | #N/A | Acidobacteria | Acidobacteria_Gp17 | Acidobacteria_Gp17_unclassified | Acidobacteria_Gp17_unclassified | Gp17 |
| Otu64621 | rare | unclassified | unclassified | unclassified | unclassified | unclassified |
| Otu61308 | abundant | Bacteroidetes | Sphingobacteriia | Sphingobacteriales | Chitinophagaceae | Ferruginibacter |
| Otu61328 | abundant | Bacteroidetes | Sphingobacteriia | Sphingobacteriales | Chitinophagaceae | Ferruginibacter |
| Otu2560 | abundant | Acidobacteria | Acidobacteria_Gp4 | Acidobacteria_Gp4_unclassified | Acidobacteria_Gp4_unclassified | Gp4 |
| Otu61290 | abundant | Bacteroidetes | Sphingobacteriia | Sphingobacteriales | Chitinophagaceae | Terrimonas |
| Otu711 | abundant | Proteobacteria | Alphaproteobacteria | Rhizobiales | unclassified | unclassified |
| Otu6625 | #N/A | Chloroflexi | Thermomicrobia | Sphaerobacterales | Sphaerobacteraceae | Nitrolancea |
| Under |  | phylum | class | order | family | genus |
| Otu44389 | #N/A | Proteobacteria | Alphaproteobacteria | Rhizobiales | Methylobacteriaceae | Microvirga |
| Otu325 | abundant | Acidobacteria | Acidobacteria_Gp6 | Acidobacteria_Gp6_unclassified | Acidobacteria_Gp6_unclassified | Gp6 |
| Otu6491 | #N/A | Planctomycetes | Planctomycetia | Planctomycetales | Planctomycetaceae | unclassified |
| Otu44356 | #N/A | Acidobacteria | Acidobacteria_Gp3 | Acidobacteria_Gp3_unclassified | Acidobacteria_Gp3_unclassified | Gp3 |
| Otu2560 | abundant | Acidobacteria | Acidobacteria_Gp4 | Acidobacteria_Gp4_unclassified | Acidobacteria_Gp4_unclassified | Gp4 |
| Otu6503 | abundant | Chloroflexi | Anaerolineae | Anaerolineales | Anaerolineaceae | unclassified |
| Otu6523 | abundant | Proteobacteria | Deltaproteobacteria | unclassified | unclassified | unclassified |
| Otu61307 | abundant | Bacteroidetes | Sphingobacteriia | Sphingobacteriales | Chitinophagaceae | Flavitalea |
| Otu2654 | #N/A | Actinobacteria | Actinobacteria | Actinomycetales | Streptomycetaceae | Streptomyces |
| Otu6480 | abundant | Bacteroidetes | Sphingobacteriia | Sphingobacteriales | Chitinophagaceae | Terrimonas |
